# From Cysteamine to Pepstatin A: Targeting Cathepsin D To Improve Bone Health in Cystinosis

**DOI:** 10.7150/ijbs.135791

**Published:** 2026-08-11

**Authors:** Giulia Battafarano, Michela Rossi, Olivia Pagliarosi, Laura Di Giuseppe, Jacopo Di Gregorio, Sara Terreri, Cristiano De Stefanis, Marco Pezzullo, Gianna Di Giovamberardino, Pamela Vernocchi, Valeria Marzano, Lorenza Putignani, Jessica D'Amico, Fiorella Piemonte, Anna Taranta, Francesco Emma, Andrea Del Fattore

**Affiliations:** 1 Bone Physiopathology Research Unit, Bambino Gesù Children's Hospital, IRCCS, Rome, Italy;; 2 Department of Biotechnological and Applied Clinical Sciences, University of L'Aquila, Italy;; 3 Research laboratories, Bambino Gesù Children's Hospital, IRCCS, Rome, Italy;; 4 Research Biobank, Bambino Gesù Children's Hospital, IRCCS, Rome, Italy;; 5 Microbiome Unit, Management and Diagnostic Innovations & Clinical Pathways Research Area, Bambino Gesù Children's Hospital, IRCCS, Rome, Italy;; 6 Unit of Microbiology and Diagnostic Immunology, and Microbiome Unit, Management and Diagnostic Innovations & Clinical Pathways Research Area, Bambino Gesù Children's Hospital, IRCCS, Rome, Italy;; 7 Department of Life Sciences, Health and Health Professions, Link Campus University, Rome, Italy;; 8 Unit of Muscular and Neurodegenerative Diseases, Bambino Gesù Children's Hospital, IRCCS, Rome, Italy;; 9 Renal Diseases Research Unit, Bambino Gesù Children's Hospital, IRCCS, Rome, Italy.

**Keywords:** Cystinosis, bone phenotype, cysteamine, Pepstatin A, Cathepsin D, Sipa1

## Abstract

Nephropathic cystinosis is a rare genetic lysosomal storage disorder caused by *loss-of-function* variants of the *CTNS* gene encoding for the lysosomal H^+^/cystine symporter cystinosin. The lack of cystinosin causes cystine accumulation in lysosomes and cell damage. Children with nephropathic cystinosis commonly exhibit skeletal abnormalities, including growth retardation, osteopenia and rickets. The only cystine-depleting drug available is cysteamine. However, several observations suggest that cysteamine, particularly at high doses, may have negative effects on the skeleton. In this study, we characterized the effects of cysteamine on bone cells and we identified Pepstatin A, a Cathepsin D-inhibitor, as a potential new therapeutic treatment to restore physiological bone remodeling in cystinosis. We observed a detrimental effect of cysteamine on bone cells inducing further impairment of osteoblast differentiation/activity and failure of cysteamine intraperitoneal injection to improve the somatic growth of pre-pubertal *ctns^-/-^* mice. Moreover, we demonstrated that Pepstatin A treatment increased bone formation *in vitro* acting on the Fibronectin/Sipa1/STAT3/JunB pathway, and rescued somatic growth and bone mass in pre-pubertal *ctns^-/-^* mice. Our results provide evidence that cysteamine negatively affects bone in cystinosis and that Cathepsin D inhibition could represent a new therapeutic approach for rescue of bone remodeling in cystinosis.

## Introduction

Cystinosis is a rare autosomal recessive disease caused by *loss-of-function* variants of the *CTNS* gene, which encodes for the cystinosin symporter 1. Cystinosin is an integral lysosomal membrane protein, responsible for the proton-coupled export of cystine from the lysosomal lumen to the cytosol 2, 3. Loss of cystinosin-dependent transport activity results in the accumulation of cystine in lysosomes, leading to crystal formation and cellular damage 4. Approximately 95% of patients suffer from the more severe form of cystinosis, termed infantile nephropathic cystinosis, which typically manifests during the first year of life with renal Fanconi syndrome, leading to excessive wasting of electrolytes and metabolites in urine 5-11.

A common complication of nephropathic cystinosis is metabolic bone disorder, which affects both the axial and appendicular skeleton. Clinical manifestations include short stature, osteomalacia, osteoporosis, rickets, bone pain and deformities 12. In infancy and early childhood, patients may exhibit *genu valgum* or *varum*, metaphyseal widening and rachitic rosary. In adulthood, cystinotic patients often develop low bone mass, cortical bone impairment and increased fracture risk, particularly in the vertebrae and long bones. Recently, Florenzano *et al.* performed systematic bone and mineral evaluations of 30 cystinotic patients and reported a reduction of bone mineral density in 46% of subjects and bone fractures in 27% of patients 9.

The pathogenesis of cystinosis-associated bone disorder is multifactorial. It is largely related to renal phosphate wasting and low 1,25(OH)_2_ Vitamin D levels secondary to renal Fanconi syndrome during early childhood and, later in life, to mineral bone disorder associated with chronic kidney disease (CKD-MBD) 13. Other contributing factors include malnutrition and copper deficiency, hormonal disturbances such as hypoparathyroidism, hypothyroidism, hypogonadism and alterations in the GH/IGF-1 and insulin pathways. Additionally, myopathy may contribute to bone disorder in cystinosis, given the functional interplay between bone and muscle 14. Importantly, we have previously reported an intrinsic bone defect in 1-month-old *ctns^-/-^* mice lacking of nephropathy and we demonstrated that cystinosin deficiency *per se* alters bone remodeling activity 15.

Cysteamine (β-mercaptoethylamine) has represented a major advance in the treatment of cystinosis as it allows lysosomal cystine-depletion. The most widely used formulation is cysteamine bitartrate which was introduced on the market in the 1990's. Early initiation and good adherence to cysteamine therapy significantly improves renal outcomes 16, 17.

However, signs of cysteamine toxicity including skin abnormalities and bone deformities, despite normal serum levels of Vitamin D, phosphate, and calcium, have been reported in patients with cystinosis treated with doses ranging from 0.90 to 1.90 g/m^2^/day. Although these effects may have been associated with copper deficiency 18, the underlying molecular mechanisms of cysteamine-induced toxicity have not been clearly demonstrated.

An inhibitory effect of high dose of cysteamine on osteoblast proliferation and osteoclast function has already been investigated in cultures of control bone cells 19. Interestingly, Claramunt-Taberner *et al.* reported a significant increase in the impairment of bone resorption when patient-derived osteoclast cultures were exposed to 200 µM of cysteamine 19.

Herein, we report the *in vitro* and *in vivo* detrimental effects of cysteamine on osteoblasts and bone phenotype of *ctns^-/-^* mice, and characterize the proteomic alterations occurring in *ctns^-/-^* osteoblasts, revealing high levels of Cathepsin D (CtsD). Furthermore, we show that the treatment with Pepstatin A, a CtsD inhibitor, restores physiological bone remodeling activity in *ctns* knock-out (KO) mice, as demonstrated by our *in vitro* and *in vivo* experiments.

## Materials and Methods

### Animals

All animal procedures were conducted in accordance with national and international guidelines (EEC Council Directive 86/609; Italian Legislative Decree 116/92; NIH guide for the Care and Use of Laboratory Animals) and under Ministry approval (Protocol number 443/2021-PR). For cysteamine study, 4-day-old *ctns*^-/-^ (KO) mice were intraperitoneally injected with 120 mg/kg cysteamine everyday 20 (Sigma-Aldrich, MO, USA) (n=4) or vehicle (n=5) as wild-type (WT) animals (n=5). To evaluate the *in vivo* effects of Pepstatin A, 4-day-old KO animals were treated five days *per* week with intraperitoneal injections of 2.5 mg/kg Pepstatin A (Sigma-Aldrich, MO, USA) (n=6), or vehicle (n=7) as WT animals (n=6). At the 30^th^ day of life,* ctns*^-/-^ and wild-type mice were sacrificed by cervical dislocation and long bones, kidney and other vital organs were collected for structural and histological analyses.

### Osteoblast cultures

Osteoblasts were isolated by sequential digestion of neonatal calvaria or long bones (tibia and femur) extracted from untreated WT and KO mice using *Clostridium histolyticum* collagenase (Sigma-Aldrich, MO, USA) and trypsin (GIBCO, Thermo Fisher Scientific, MA, USA) at 37 °C (15, 30, and 45 minutes, in gentle agitation). Cells were cultured in Dulbecco's modified Eagle's medium (DMEM) supplemented with 10% Fetal Bovine Serum (FBS), trypsinized, and plated according to the experimental protocol. After 48h of treatment with 100 µM of cysteamine, 10 µM Pepstatin A, or phosphate buffer and DMSO vehicles as respective controls, alkaline phosphatase (ALP) activity was assessed by ALP 86C-1KT (Sigma-Aldrich, MO, USA), according to manufacturer's instruction.

### Cystine measurement

Osteoblasts were sonicated in the presence of 10 mM N-ethylmaleimide. The protein fraction was precipitated by the addition of 10% 5-sulfosalicylic acid and protein content was determined by Pierce Bicinchoninic Acid protein assay. Cystine levels were measured by High Performance Liquid Chromatography (HPLC) as previously described in Pastore *et al.* 21.

### Real Time RT-PCR

Total RNA was extracted from osteoblast cell culture or tissues using the Trizol® (Sigma-Aldrich, MO, USA) procedure. One microgram of RNA was reverse transcribed using the SensiFast cDNA Synthesis Kit (Bio Line, Italy) and the equivalent of 50 ng of cDNA was used for Real Time PCR analysis. The primers used and their sequences are listed in Table 1.

### XTT Assay

Cell viability was evaluated by XTT Assay (Roche, Italy) according to the manufacturer's procedure. Cells were seeded in 96-well plates and treated for 24 hours before the addition of activated-XTT solution. After 4 hours of incubation at 37 °C and 5% CO_2_, absorbance was read at 450 nm by using a colorimetric plate reader and 650 nm wavelength was used as reference.

### Colony-Forming Unit (CFU) Assay

Total bone marrow was flushed out from long bones of 10-day-old WT and *ctns^-/-^* mice. Cells were collected after centrifugation at 300g for 3 minutes, resuspended and plated at the density of 1x10^6^/100mm dish culture in the presence of 100 µM cysteamine, 10 µM Pepstatin A or vehicles. After 10 days of culture, crystal violet and ALP stainings were performed to identify colonies.

### Mineralization Assay

Calvarial osteoblasts were cultured in DMEM plus 10% FBS, 5 mmol/L β-glycerophosphate, 50 μg/mL ascorbic acid to induce mineralization and 100 µM of cysteamine, 10 µM Pepstatin A or vehicles. After 3 weeks, Von Kossa staining was performed and the mineralized area was measured by densitometry performed on 10X original magnification images (Leica, DMi8) and analyzed by ImageJ software.

### Osteoclasts from total bone marrow cultures

Total bone marrow was flushed out from long bones of 10-day-old WT and *ctns^-/-^* mice. Cells were collected after centrifugation at 300g for 3 minutes, resuspended at the density of 2x10^6^/ml and plated in 96-well plates in culture medium supplemented with 10^-8^M 1,25(OH)_2_D according to Marino *et al.* 22. The medium was replaced the following day with fresh medium containing either 100 µM cysteamine, 10 µM Pepstatin A or vehicles. After 7 days of culture, cytochemical TRAcP (Tartrate Resistant Acid Phosphatase) and Hoechst stainings (Sigma-Aldrich, MO, USA) were performed. TRAcP-positive cells with 3 or more nuclei were considered as osteoclasts and were counted.

### Histological analysis

Organs and bones extracted from animals were fixed in 4% formaldehyde in 0.1 M phosphate buffer, pH 7.2 and dehydrated in ethanol. Bone was decalcified using Microdec, EDTA-based (Diapath, Italy) and processed for paraffin embedding. Three μm-thick sections were stained with hematoxylin and eosin (Diapath, Italy). Bones were stained for Toluidine Blue and TRAcP (Sigma-Aldrich, MO, USA). Histomorphometric analysis was performed on 2-3 μm-thick sections using NDP.view2 Image viewing software (Hamamatsu Photonix K.K., Japan). Nomenclature, symbols and units of histomorphometric bone parameters were defined according to the guidelines of the ASBMR Histomorphometry Nomenclature Committee 23.

### Micro-CT

In order to assess trabecular bone microarchitecture and cortical bone morphology, femurs were excised, cleaned to remove soft tissues and scanned with high-resolution desktop micro-tomographic imaging system (µCT40, Scanco Medical AG, Switzerland). Image acquisition and analysis protocols were conducted according to guidelines for the use of µCT to assess bone microstructure in rodents 24, as we have previously reported 15. Briefly, scans were acquired using a 10 µm^3^ isotropic voxel size, 70 kVp peak X-ray tube intensity, 114 mA X-ray tube current, 200 ms integration time, and were subjected to Gaussian filtration and segmentation. Trabecular bone was evaluated in the distal femur metaphysis and segmented from soft-tissue using a threshold of 330 mgHA/cm^3^. Cortical bone architecture was evaluated at the femoral mid-diaphysis and segmented using a threshold of 700 mgHA/cm^3^. The measurements of trabecular bone volume fraction (BV/TV, %), trabecular thickness (Tb.Th, mm), trabecular number (Tb.N, 1/mm), trabecular separation (Tb.Sp, mm), trabecular bone mineral density (Tb.BMD, mgHA/cm^3^), connectivity density (Conn.D, 1/mm^3^) and structural model index (SMI) were performed using the Scanco Evaluation program Trabecular Morphology script. The Scanco Mid-shaft Evaluation script was used to measure total cross-sectional area (Tt.Ar, mm^2^), cortical bone area (Ct.Ar, mm^2^), medullary area (Ma.Ar, mm^2^), cortical bone area fraction (Ct.Ar/Tt.Ar, %), cortical thickness (Ct.Th, mm), cortical tissue mineral density (Ct. TMD, mgHA/cm^3^) as well as the maximum, minimum and polar moments of inertia (Imax, Imin and pMOI, mm^4^).

### Proteomic analysis

WT and KO calvarial osteoblasts were seeded for protein analysis, and after 48h were washed with cold PBS (Phosphate Buffered Saline) and lysed using radioimmunoprecipitation assay buffer (RIPA, Sigma-Aldrich, MO, USA) with protease inhibitor for 30 minutes on ice. Twenty μg of protein extract were subjected to reduction, alkylation and trypsin digestion according to the filter-aided sample preparation protocol 25. Briefly, protein extracts were loaded on Microcon-10 kDa Centrifugal Filter Unit with Ultracel-10 membrane (Merck, MA, USA) in the presence of 8 M urea and 100 mM Tris-HCl, pH 8.5; disulfide bonds were reduced and alkylated with 8 mM dithiothreitol and 50 mM iodacetammide; proteins were digested with 0.5 μg of sequencing grade trypsin (Promega, Italy) at 37 °C in 50 mM ammonium bicarbonate buffer pH 8.0 overnight. Peptides were eluted from the Microcon, speedvac dried, and resuspended in a water solution containing 2% acetonitrile (ACN) and 0.1% formic acid (FA). Three micrograms of these samples were subjected to nano-HPLC-ElectroSpray Ionization-tandem Mass spectrometry (nano-HPLC-ESI-MS/MS) analysis. This analysis was performed on an Eksigent Ekspert Nano LC400 system (Sciex, ON, Canada), which was directly coupled to a TripleTOF 5600+ (Sciex) with a nanoESI source (NANOSpray III, Sciex), as already described 26. For each of three biological replicates, three MS/MS raw data were obtained.

Protein identification and label-free quantification (LFQ) was performed using MaxQuant software (version 1.5.8.5) as previously described by Pepe *et al.* 26. The search was conducted using the *Mus musculus* UniProtKB/Swiss-Prot Protein Knowledgebase (release 2018_04, containing 16,966 proteins). In order to identify differentially expressed proteins (DEPs) between the two experimental groups, Student's t-test univariate analysis was performed on LFQ intensities with the Perseus software (version 1.5.8.5) of the MaxQuant computational platform 27. Results with permutation-based False Discovery rate (FDR) < 0.05 were considered to be statistically significant. The hierarchical cluster analysis, visualized by heatmap, was based on the LFQ protein intensity abundances applying a z-score transformation, computing the distance function by Euclidean correlation and the linkage by Ward clustering method (MetaboAnalyst version 4.0) 28.

DEPs were analyzed using the Protein ANalysis Through Evolutionary Relationships (PANTHER) Overrepresentation Test (Released 2018-11-13) 29 using the annotation sources of Gene Ontology (GO) database (released 2019-01-01), PANTHER knowledgebase (version 14.0, released 2018-12-03), and Reactome (version 65, released 2018-06-12), employing a Fisher's Exact test with an FDR for multiple test correction.

The second dataset derived from Pepstatin A or vehicle-treated *ctns*^-/-^ osteoblasts (five biological replicates for each condition) was analyzed by nano-HPLC-ESI-MS/MS by applying a Data-Independent Acquisition (DIA) strategy 30. Proteins were digested with trypsin at a protein-to-enzyme ratio of 40:1, and the resulting enzymatic peptides were desalted using a Strata X column and vacuum-dried. Dried peptide samples were reconstituted in 2% ACN, 0.1% FA. Chromatographic separation and MS spectrometry acquisition were performed on UltiMate3000 RSLCnano system directly coupled to an Orbitrap Exploris 480 (Thermo Fisher Scientific, San Jose, CA). Peptides were first trapped and desalted on a trap column, then separated on an *in-house* packed C18 analytical column (150 μm i.d., 1.8 μm particle size, 35 cm length) at a flow rate of 500 nL/min using two different gradients: a) one of 180 min for data dependent acquisition (DDA) and b) the second of 120 min, for the DIA. DDA data was identified by MaxQuant, and identification results were used for spectral library construction to be used for the DIA data; mProphet algorithm was used to complete analytical quality control, thus obtaining a large number of reliable quantitative results. Quantification of proteins was performed using the MSstats software package 31. Based on predefined comparison groups and a linear mixed-effects model, proteins meeting both filtration criteria -absolute fold change > 2 and p-value adjusted for multiple comparisons using the false discovery rate (FDR) according to the Benjamini-Hochberg procedure < 0.05- were considered significantly DEPs.

### Western blotting and cell fractionation analysis

Proteins were resolved by SDS-Polyacrylamide gel electrophoresis (PAGE) and transferred to nitrocellulose or PVDF membranes (Biorad Laboratories, CA, USA). Blots were probed with primary antibodies against Cathepsin D (1:3000, AB75852 Abcam, UK), Sipa1 (1:1000, A12253 AbClonal, MA, USA), Fibronectin (1:200, sc-9068 SantaCruz Biotech, TX, USA), p-STAT3 (1:1000, 9131 Cell Signaling Technology, MA, USA), STAT3 (1:1000, 12640 Cell Signaling Technology, MA, USA), JunB (1:1000, BK3753S Cell Signaling Technology, MA, USA), β-Actin (1:10000, 5125 Cell Signaling Technology, MA, USA), β-Tubulin (1:5000, MABS156627 Immunological Sciences, Italy), Na^+^/K^+^ ATPase (1:3000, AB76020 Abcam, UK) and Histone H3 (1:2000, sc-8654-R SantaCruz Biotech, TX, USA). Membranes were incubated overnight at 4 °C, washed with Tris-Buffered Saline with Tween 20 (TBST) and incubated with the appropriate horseradish peroxidase (HRP)-conjugated secondary antibody (1:5000, Biorad Laboratories, CA, USA) at room temperature for 1h. Protein bands were revealed by ECL detection (Biorad Laboratories, CA, USA) according to the manufacturer's instruction and revealed by Chemidoc Touch Imaging System (Biorad Laboratories, CA, USA). Densitometric analysis was performed by ImageJ software.

### Statistical analyses

Data were expressed as mean±SEM (Standard Error of Mean) of at least three independent experiments or four animals *per* group. Comparisons among three groups were performed using one-way ANOVA followed by Tukey's or Šídák's multiple comparisons test. For the analysis of cathepsin D activity assay with only two experimental groups, values were compared by unpaired student's *t* test. Results with p < 0.05 were considered statistically significant. Statistical analyses were performed using GraphPad Prism 10.

## Results

### Cysteamine treatment affects *ctns^-/-^* osteoblasts *in vitro* and *in vivo*

**Figure 1:**
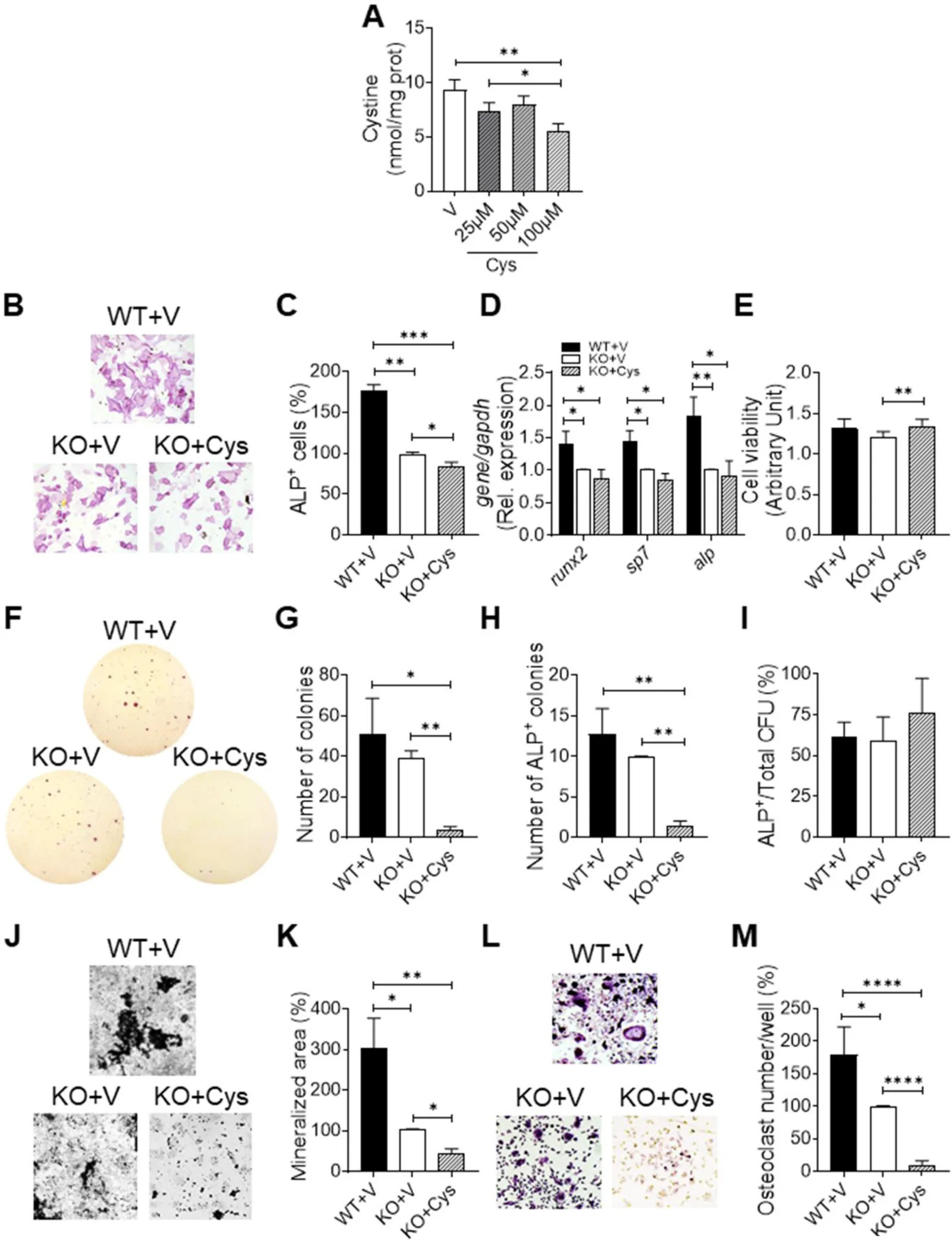
***In vitro* effect of cysteamine on *ctns^-/-^* osteoblasts**. **(A)** HPLC measurement of cystine levels in osteoblasts isolated from calvariae of KO mice and cultured for 48h in the presence of cysteamine (Cys) at the reported concentrations or vehicle (V). **(B-F)** Effects of 100 µM cysteamine treatment on calvarial osteoblasts. **(B)** Representative pictures (Original magnification 10X) and **(C)** densitometric analysis of ALP staining. **(D)** Real Time RT-PCR expression analysis of *runx2*, *sp7* and* alp*. **E)** Cell viability assessed by XTT assay. **(F-I)** Colony-Forming Unit (CFU) assay with total bone marrow cells isolated from wild-type and KO mice and treated with vehicle or 100 µM Cysteamine. **(F)** Representative pictures of CFU obtained after 10 days of culture and identified by Crystal Violet staining and **(G-I)** quantification of total and ALP positive colonies. **(J)** Representative images of Von Kossa staining showing mineralized matrix (in black) produced by osteoblasts (original magnification 10X) and **(K)** quantification of mineralized area. **(L-M)** Total bone marrow was flushed out from mice, stimulated with 1,25 Dihydroxyvitamin D_3_ and cultured for 7 days in the presence of 100 µM cysteamine or vehicle. **(L)** Representative pictures of TRAcP (Tartrate Resistant Acid Phosphatase) staining showing TRAcP-positive multinucleated osteoclasts. Original magnification 10X. **(M)** Quantification of osteoclast number. Data are reported as mean±SEM of at least 3 independent cell cultures. *p < 0.05, **p < 0.01, ***p < 0.001, ****p < 0.0001.

To understand the direct effect of cysteamine on bone remodeling in cystinosis, we performed an *in vitro* study of *ctns^-/-^* bone cells treated with cysteamine. Given the relevant role of osteoblasts during growth, we first determined the cysteamine concentration that could reduce cystine levels in osteoblasts isolated from calvariae of *ctns^-/-^* mice. As shown in Figure 1A, 100 µM cysteamine significantly decreased intralysosomal cystine accumulation. This concentration was therefore used in all subsequent *in vitro* experiments. Overall, ALP positivity showed a 45% decrease in KO cells, compared to wild-type cells. Cysteamine treatment induced a moderate but significant further reduction of osteoblast ALP positivity (Figure 1B-C), but no significant changes in the gene expression of osteoblast differentiation and activity markers (Figure 1D), compared to vehicle-treated KO cells. Interestingly, 24h of cysteamine treatment of *ctns^-/-^* calvarial osteoblasts slightly increased their viability compared to vehicle treatment (Figure 1E). The effects of cysteamine on ALP activity and cell viability were confirmed on osteoblasts isolated from long bones (Supplementary Figure 1). To further assess the impact of cysteamine on osteoblast differentiation, we evaluated the ability of bone marrow-derived stromal cells to form ALP^+^ colonies with a Colony-Forming Unit (CFU) assay. As shown in Figure 1F-I, incubation of bone marrow cells with cysteamine significantly impaired self-renewal and differentiation ability of KO cells into ALP-positive CFU. Moreover, cysteamine treatment further impaired *ctns^-/-^* osteoblast mineralization ability as shown by Von Kossa staining revealing a 60% reduction of mineralized area in cysteamine-treated *ctns^-/-^* cultures, compared to vehicle-treated cultures (Figure 1J-K).

To evaluate the effect of cysteamine treatment on the ability of bone marrow stromal cells to support osteoclast differentiation, we isolated bone marrow cells from long bones of WT and KO mice and cultured them in the presence of Vitamin D3. Consistent with our previous results 15, we observed a significant impairment (~50%) of osteoclast formation in cultures obtained from KO mice-derived cells and a further reduction of osteoclastogenesis following cysteamine treatment (Figure 1L-M).

**Figure 2:**
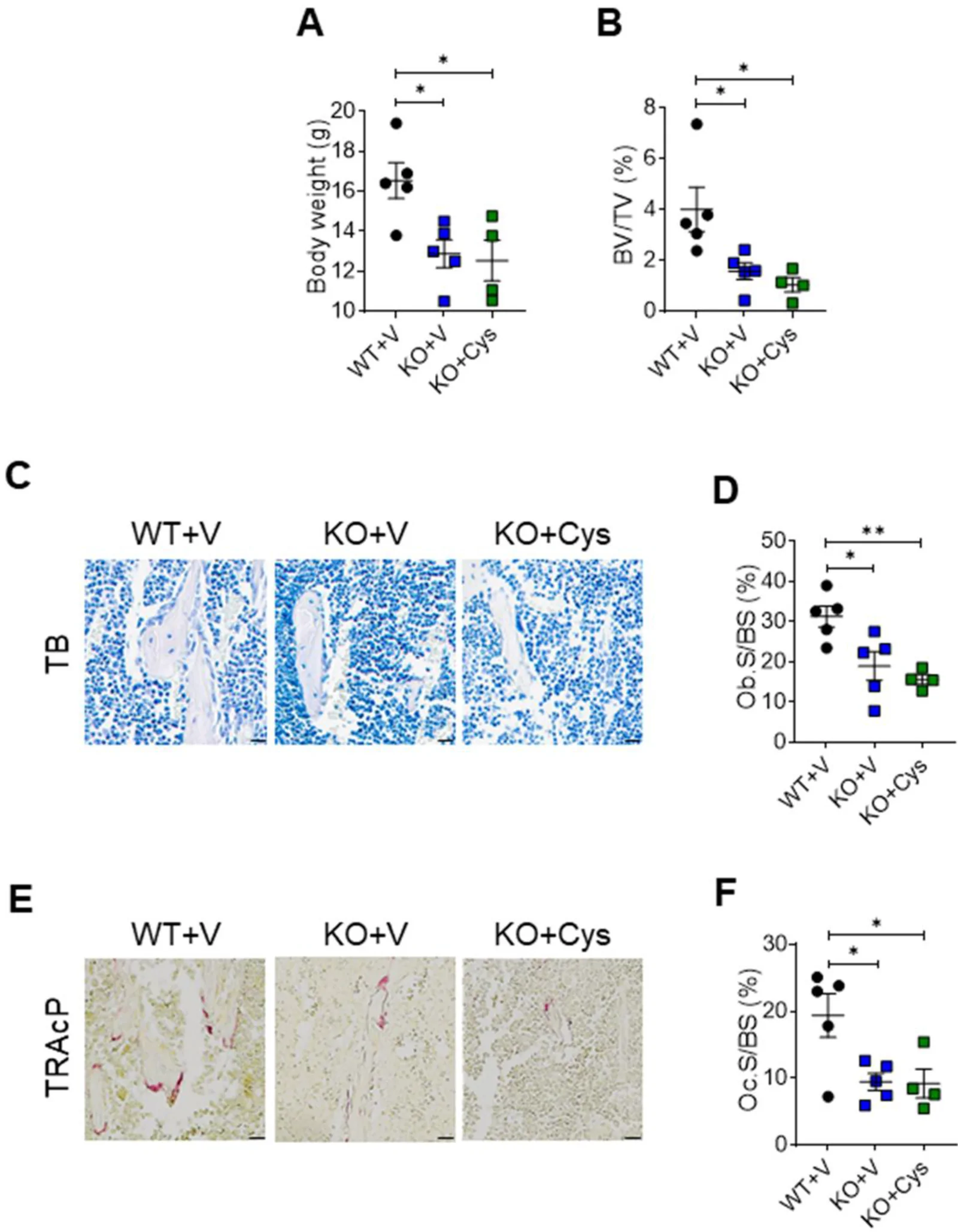
** Effect of cysteamine treatment on pre-pubertal somatic growth of *ctns^-/-^* (KO) mice**. WT and KO mice were daily treated with vehicle (V) or cysteamine (Cys, 120 µg/g/day). **(A)** Body weight of *ctns^-/-^* mice at day 30. **(B)** Histomorphometric analysis of trabecular bone/total volume (BV/TV) in tibia of WT and KO mice treated with cysteamine or vehicle. **(C)** Representative images of Toluidine Blue stained semithin section (original magnification 20X, scale bar 300 µm) and **(D)** quantification of trabecular bone surface covered by osteoblasts (Ob.S/BS). **(E)** Representative pictures of TRAcP staining (original magnification 20X, scale bar 200 µm) and **(F)** quantification of bone surface covered by osteoclasts (Oc.S/BS) of WT and KO animals. Values are reported as mean±SEM. *p < 0.05, **p < 0.01.

Interestingly, the treatment of animals from the 4^th^ to the 30^th^ day of life with intraperitoneal injection of cysteamine, at a dose of 120 mg/kg/day previously tested in mice Reda *et al.* 20, failed to improve body weight gain and bone mass of *ctns^-/-^* mice compared to vehicle-treated KO animals (Figure 2A-B), suggesting the inability of cysteamine to rescue the bone remodeling defect. Moreover, spleen, liver and kidney weights were consistent with the body weight and no evidence of cysteamine-induced signs of inflammation, degeneration, necrosis, fibrosis and structural damage were reported by histological analysis (Supplementary Figure 2). As we have already reported 15, histomorphometric analysis of osteoblast parameters showed a 40% decrease of osteoblast surface/bone surface in transgenic mice, and a trend of further reduction was observed after cysteamine treatment (Figure 2C-D). Histochemical analysis of the osteoclast-specific marker TRAcP revealed a 51% reduction of TRAcP-positive cells lining the bone trabeculae of the proximal spongiosa in *ctns^-/-^* mice. Cysteamine treatment did not modify osteoclast surface/bone surface in transgenic animals (Figure 2E-F).

### Evaluation of Cathepsin D expression in* ctns^-/-^* osteoblasts

**Figure 3:**
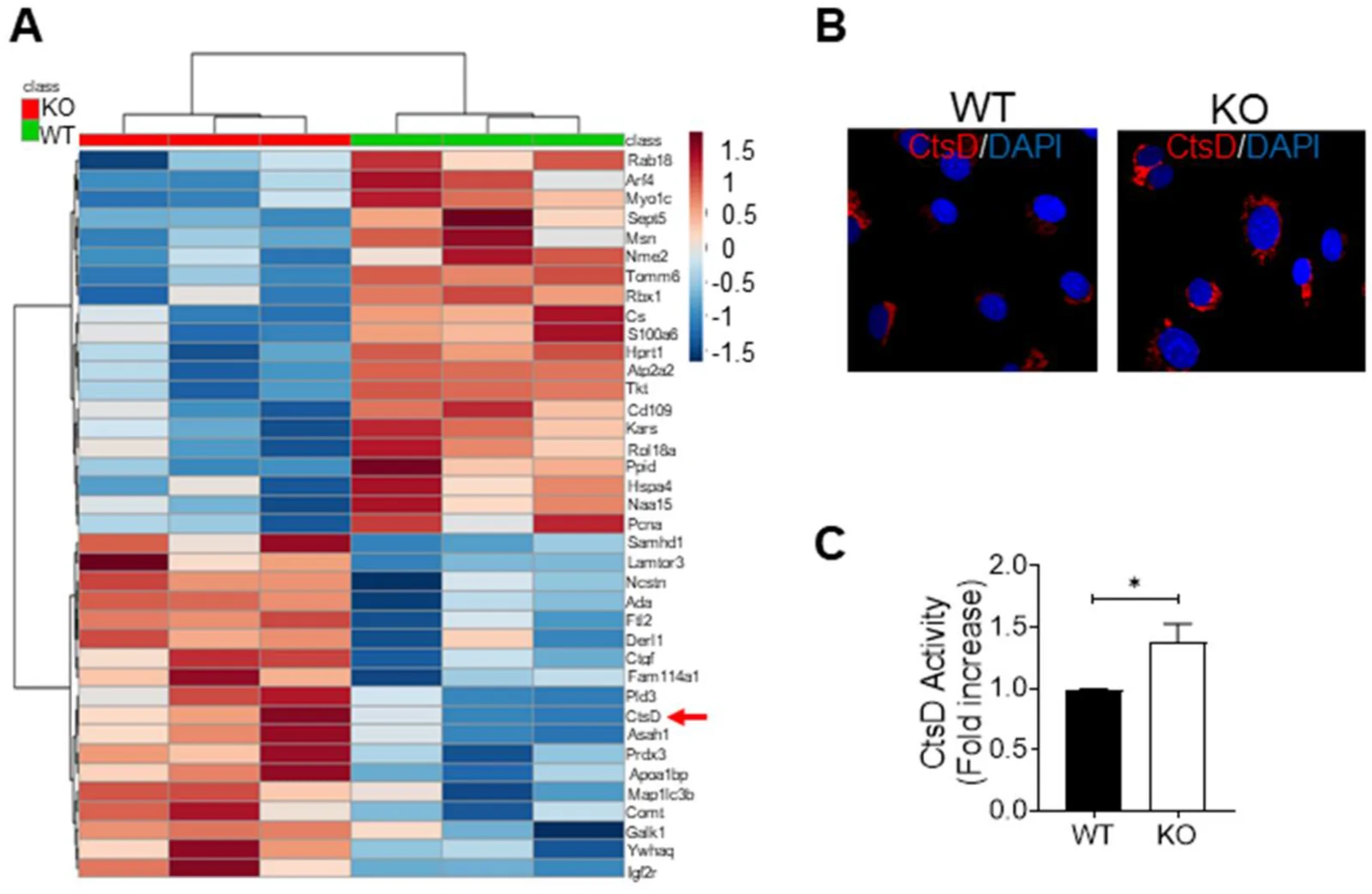
** Proteomic analysis and Cathepsin D expression in wild-type and *ctns^-/-^* osteoblasts. (A)** Hierarchical clustering of the 38 differentially expressed proteins (DEPs) between KO and WT mice osteoblasts. A heatmap, based on normalized protein abundances and subjected to a z-score transformation, was utilized to visualize color-coded hierarchical clusters from KO (red color) and WT (green color) osteoblasts. The dendrogram above the heat map, representing the distance between samples, demonstrated similarity among samples of each mice group (class). The top right heat map color legend displays the range of the scaled DEPs abundance values, ranging from -1.5 to +1.5. The red arrow indicates the lysosomal protein Cathepsin D (CtsD), which is one of the DEPs with the highest identification score that has been identified as over-expressed. **(B)** Confocal microscopy analysis of Cathepsin D expression. **(C)** Fluorescence activity analysis of Cathepsin D in WT and KO osteoblasts. Values are reported as mean±SEM of at least 7 independent cell cultures. *p < 0.05.

To investigate molecular changes in *ctns^-/-^* osteoblasts and to identify potential therapeutic strategies to correct bone defects in cystinosis, we conducted an untargeted bottom-up shotgun mass spectrometry (MS)-based proteomic analysis. A total of 38 proteins were found to be differentially expressed when comparing KO and WT osteoblasts (Figure 3A and Supplementary Table S1). In depth analysis of over- and under-represented functions associated to the DEPs, we identified an elevated number of proteins involved in catalytic activity (GO:0003824, 23 DEPs) and protein binding (GO:0005515, 28 DEPs) molecular functions, as well as proteins localized to the lysosomes (GO:0005764, 7 DEPs) (Supplementary Table S2). Among the differentially expressed proteins, the lysosomal aspartic protease Cathepsin D (CtsD) was significantly upregulated in KO osteoblasts, exhibiting a high identification score (Supplementary Table S1 and Figure 3A). Increased CtsD expression in *ctns^-/-^* osteoblasts was confirmed by confocal immufluorescence analysis (Figure 3B). To evaluate whether the increased expression of Cathepsin D was also associated with enhanced proteolytic ability, CtsD activity was assessed using a CtsD substrate sequence GKPILFFRLK(Dnp)-D-R-NH2) conjugated to MCA (7-Methoxycoumarin-4-acetic acid). The fluorescence analysis revealed a 1.38-fold increase of CtsD proteolytic ability in KO cells compared to WT osteoblasts (Figure 3C).

### Cathepsin D inhibition improves the activity of *ctns^-/-^* osteoblasts *in vitro*

**Figure 4:**
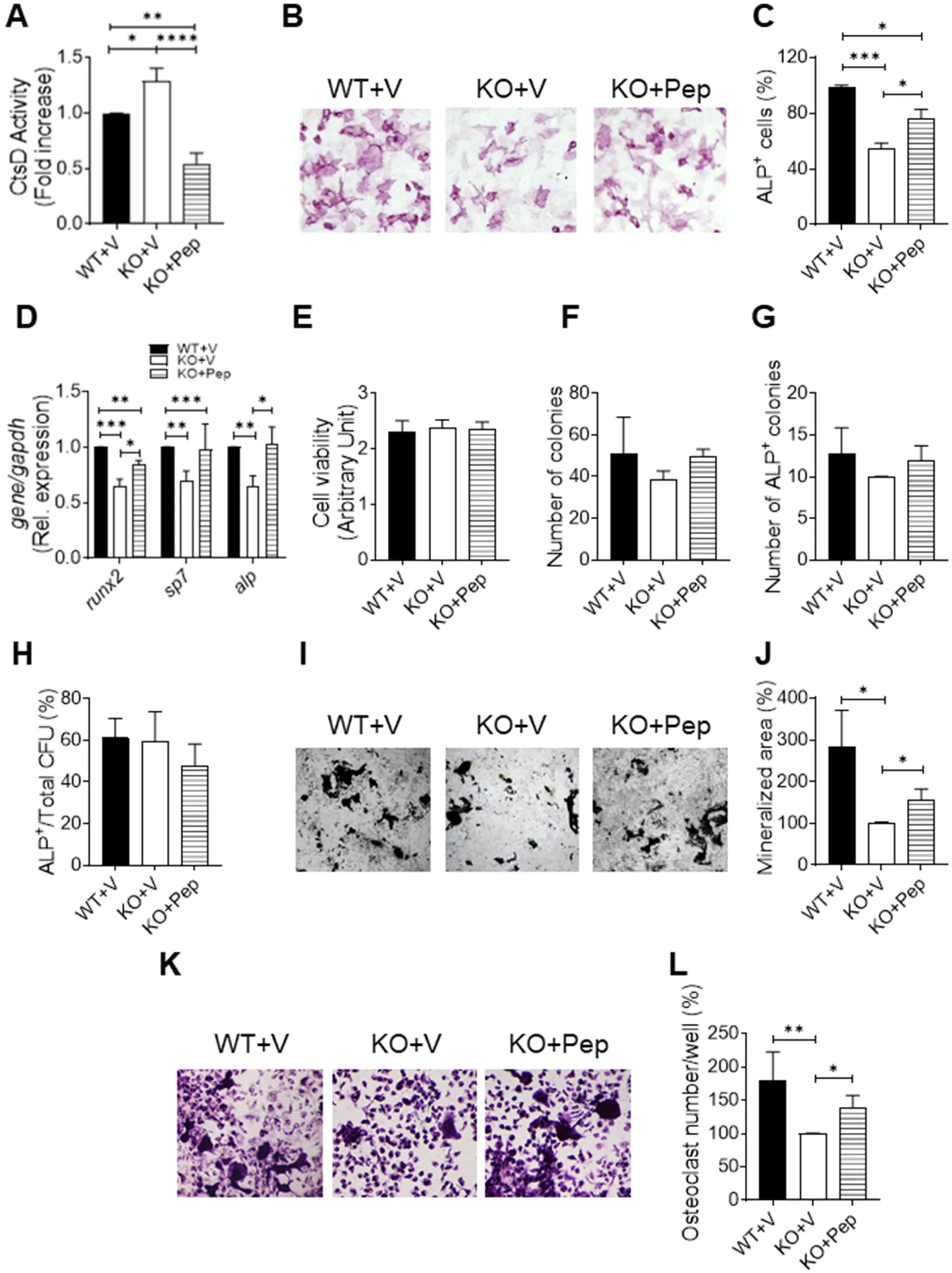
** Pepstatin A treatment of KO osteoblasts**. **(A-E)** Osteoblasts from calvaria of wild-type and KO mice were treated with vehicle or 10 µM Pepstatin A. **(A)** Cathepsin D protein activity. **(B)** Representative pictures of ALP staining (Original magnification 10X) and **C)** densitometric analysis. **(D)** Real Time RT-PCR expression analysis of *runx2*, *sp7* and *alp*.** (E)** Cell viability after 24h of treatment. **(F-H)** Quantification of total and ALP positive colonies obtained by treatment of total bone marrow cells isolated from wild-type and KO mice with vehicle or 10 µM Pepstatin A. **(I)** Representative pictures of Von Kossa staining of calvarial osteoblasts. Original magnification 10X. **(J)** Percentage of mineralized area. **(K)** Representative pictures of TRAcP staining (Original magnification 10X) and **L)** quantification of osteoclast number of total bone marrow cultures. Values are reported as mean±SEM of at least 3 independent cell cultures. *p<0.05, **p<0.01, ***p<0.001, ****p<0.0001.

To test if Cathepsin D may represent a therapeutic target, we treated *ctns^-/-^* osteoblasts with 10 µM Cathepsin D-inhibitor Pepstatin A (Pep). As expected, the treatment determined a 58% decrease of CtsD protein activity in KO cells (Figure 4A). Moreover, although Pep treatment was not able to reduce intracellular cystine content (nmol/mg protein in osteoblasts, WT+Vehicle: 0.60±0.29; KO+Vehicle: 5.16±0.38***; KO+Pep: 4.32±0.28***; ***p < 0.001 *vs* WT+Vehicle), it stimulated osteoblasts as shown by ALP staining (Figure 4B-C) and by enhanced *alp* and *runx2* (Runt-related transcription factor 2) gene expression and a trend of increase of *sp7* (Osterix) (Figure 4D). Pepstatin A treatment did not affect cell viability of *ctns^-/-^* osteoblasts (Figure 4E), nor self-renewal and osteogenic differentiation of bone marrow stromal cells (Figure 4F-H). However, it significantly improved mineralization activity in KO cells (1.57 fold, p < 0.05) compared to vehicle treatment (Figure 4I-J). Interestingly, Pep treatment rescued the ability of bone marrow stromal cells isolated from *ctns^-/-^* mice to support osteoclast differentiation (Figure 4K-L), suggesting that CtsD inhibition could restore the physiological bone remodeling activity in cystinosis.

### Rescue of the growth defect in *ctns^-/-^* mice treated with Pepstatin A

**Figure 5:**
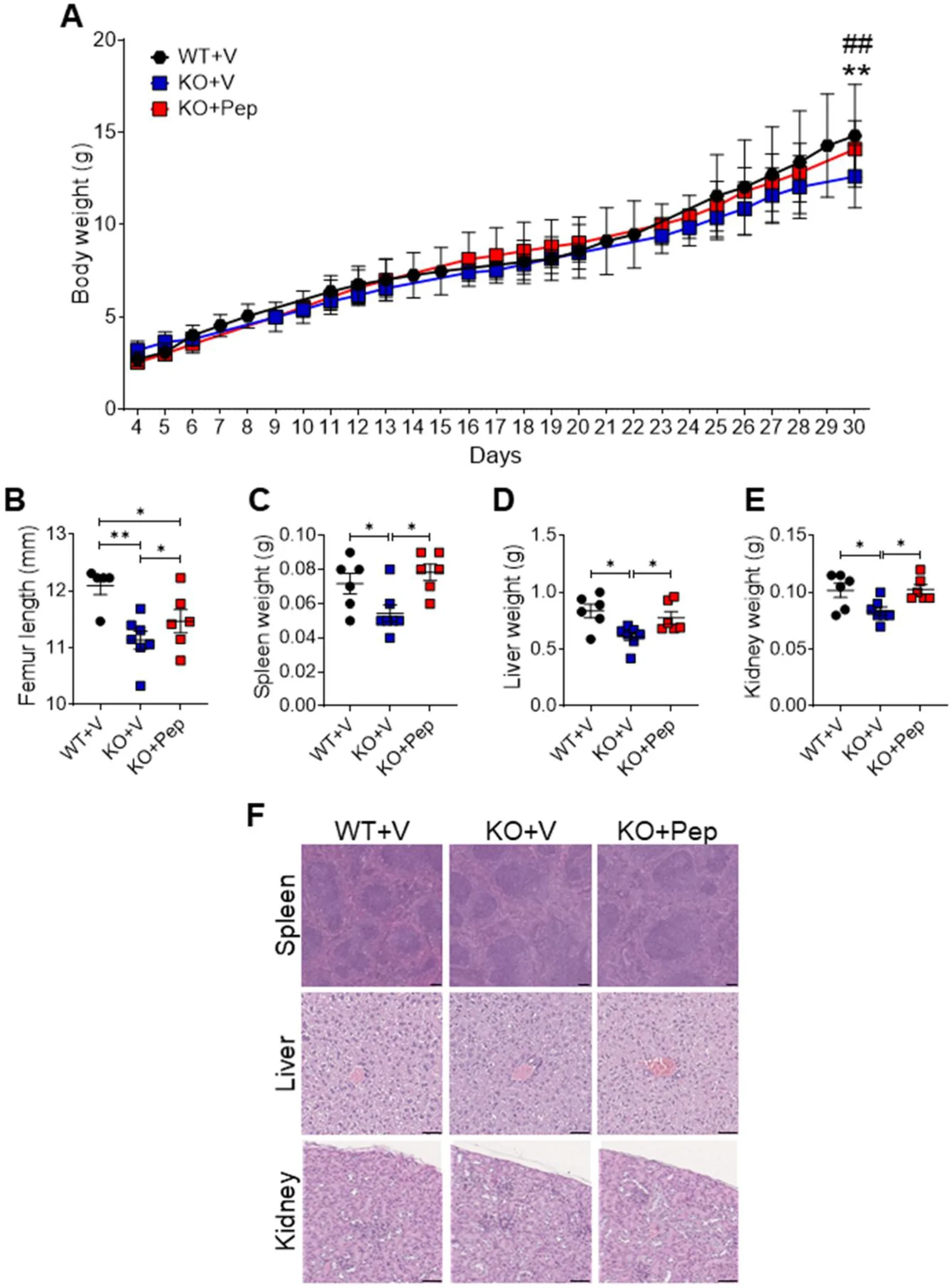
** Effect of Pepstatin A treatment *in vivo*.** WT and KO mice were treated with vehicle (V) or Pepstatin A (Pep, 2.5 mg/kg/day).** (A-B)** Analysis of somatic growth. **(A)** Body weight. Data are reported as mean±SEM. ^**^p<0.01 *vs* WT+Vehicle; ^##^p<0.01 *vs* KO+Vehicle. **(B)** Femur length. **(C-E)** Weight of **(C)** spleen, **(D)** liver and **(E)** kidney of wild-type and *ctns^-/-^* mice treated with vehicle or with Pepstatin A. In **(B-E)** data are reported as mean±SEM. *p < 0.05, **p < 0.01. **(F)** Representative pictures of Hematoxylin/eosin staining of spleen (original magnification 5X, scale bar 500µm), liver and kidney (original magnification 10X, Scale bar 500 µm).

To investigate the effects of Pepstatin A treatment on somatic growth and bone phenotype of *ctns^-/-^* mice, KO mice were injected intraperitoneally with 2.5 mg/kg Pepstatin A, five days *per* week, as previously described by Rowe *et al*. 32, from postnatal day 4 to day 30. Pepstatin A treatment completely prevented the growth defect, as shown by the recovery of body weight and the increase of femur length in KO mice (Figure 5A-B).

Measurement of spleen, liver and kidney weights were consistent with the recovery of normal body growth (Figure 5C-E). Macroscopic and histological analyses of vital organs revealed no evidence of Pepstatin A-induced toxicity (Figure 5F).

### Pepstatin A rescues the bone phenotype of *ctns^-/-^* mice

**Figure 6:**
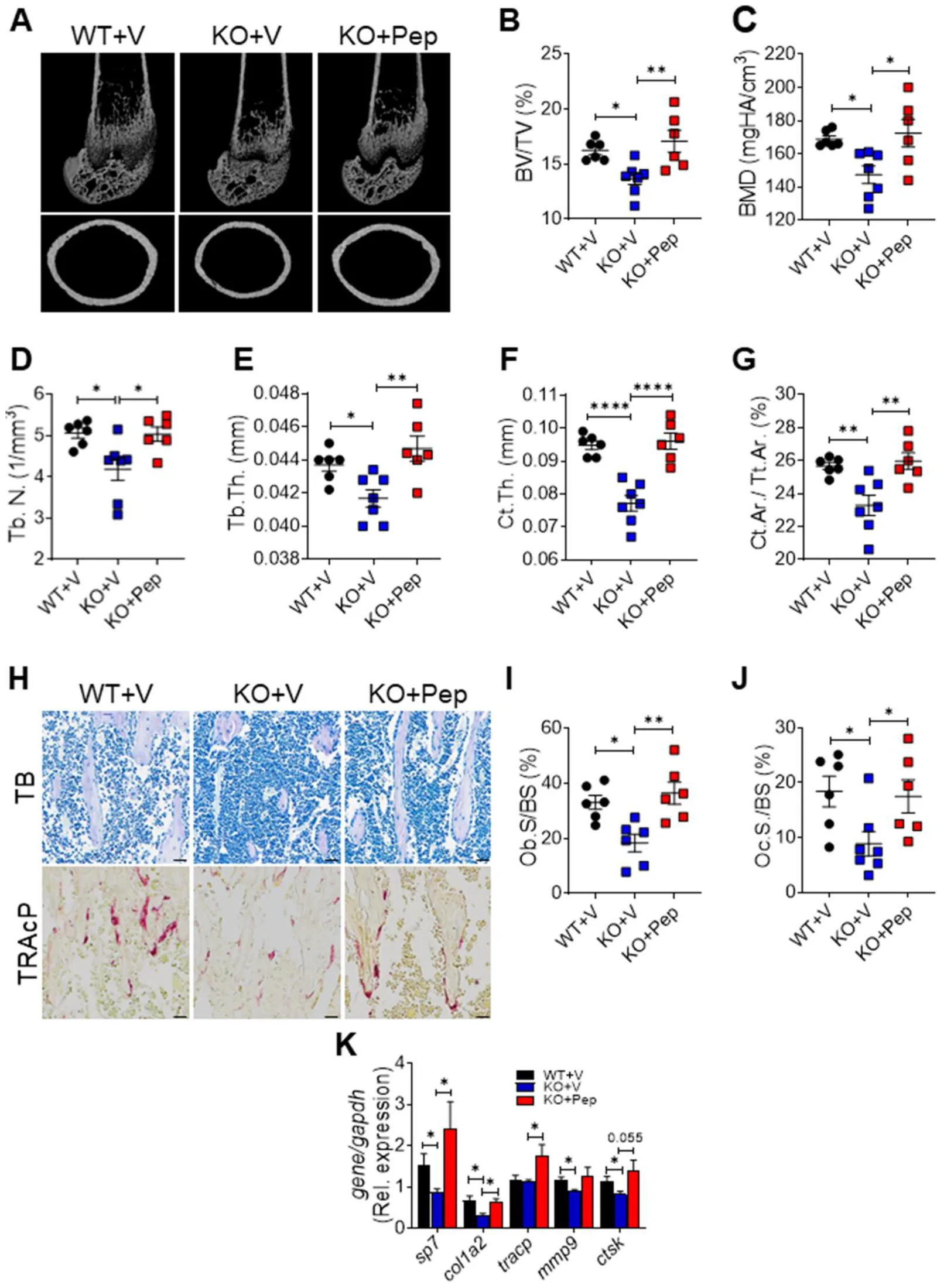
** Effect of Pepstatin A on bone phenotype. (A)** Microcomputed tomography analysis of 1-month-old wild-type (WT) and cystinotic (KO) mice treated with vehicle or Pepstatin A. Representative µCT images of *upper panels*) distal femurs and *lower panels*) mid-diaphysis of femurs. **(B)** Histomorphometric analysis of Bone Volume/Total Volume (BV/TV). **(C)** Analysis of Bone Mineral Density (BMD). Histomorphometric evaluation of **(D)** trabecular number (Tb.N), **(E)** trabecular thickness (Tb.Th), **(F)** cortical thickness (Ct.Th) and **(G)** cortical bone area/Total Area (Ct.Ar./Tt.Ar.). **(H)** Representative images of *upper panels*) Toluidine Blue and *lower panels*) TRAcP stained semithin sections of tibia. Original magnification 20X. Scale bar 500 µm and 200 µm, respectively. **(I-J)** Histomorphometric analysis of trabecular bone surface covered by **(I)** osteoblasts (Ob.S/BS) and **(J)** osteoclasts (Oc.S/BS) in tibia spongiosa of WT and KO animals. **(K)** Gene expression of osteoblast (*sp7* and *col1a2*) and osteoclast (*tracp*, *mmp9* and *ctsk*) markers measured by Real Time RT-PCR analysis on mRNA extracted from femurs of WT and KO mice. Values are normalized versus the housekeeping gene *gapdh*. Results are reported as mean±SEM. *p < 0.05, **p < 0.01, ****p < 0.0001.

We next evaluated whether Pepstatin A treatment affected the skeletal phenotype of *ctns^-/-^* mice. Pepstatin A normalized the trabecular bone phenotype, as assessed by three-dimensional micro-computed tomography (µCT) analysis of the distal femoral metaphysis (Figure 6A-G, Table 2). Specifically, µCT analysis revealed increased trabecular bone volume (BV/TV), bone mineral density (BMD), trabecular number (Tb.N), and trabecular thickness (Tb.Th) (Figure 6A-E), as well as increased connectivity density (Conn.D) (Table 2) in KO mice receiving Pepstatin A compared to vehicle-treated KO animals, reaching values comparable to those observed in vehicle-treated WT mice. Structural model index (SMI) values were significantly higher in vehicle-treated KO bones, indicating the presence of more plate-like structures compared to WT tissues, and suggesting weaker bones. Notably, Pepstatin A treatment restored SMI values to those observed in WT mice (Table 2). Moreover, μCT analysis performed at the femoral mid-diaphysis showed increased cortical bone and thickness in Pepstatin A-treated KO mice compared to vehicle-treated animals (Figure 6A, F-G, Table 2). No significant changes were observed in cortical mineral density (Table 2). Normalization of polar moments of inertia, as well as maximum and minimum moments of inertia, was also observed in *ctns^-/-^* mice following Pepstatin A treatment (Table 2).

Histological examination of semithin tibial sections showed that Pepstatin A treatment induced a ~2-fold increase of osteoblasts lining trabecular bone surfaces in *ctns^-/-^* mice, as shown by representative pictures of toluidine blue stained sections (Figure 6H). Histomorphometric analysis revealed levels of osteoblast surface/bone surface (Ob.S/BS) in Pepstatin A-treated mice similar to those observed in wild-type animals (Figure 6I). TRAcP histochemical analysis revealed an increase of TRAcP-positive cells lining bone trabeculae in Pepstatin A-treated *ctns^-/-^* mice compared to vehicle-treated KO animals (Figure 6H). Histomorphometric analysis showed normalization of osteoclast surface/bone surface (Oc.S/BS) (Figure 6J).

Accordingly, Real Time RT-PCR expression analysis revealed a significant upregulation of genes involved in osteoblast differentiation and function (*sp7* and *col1a2,* collagen type I alpha 2 chain) and osteoclast formation (*tracp*), and a trend of increased expression of bone resorption-related genes *mmp9* (Matrix metallopeptidase 9) and *ctsk* (Cathepsin K), in femurs from Pepstatin A-treated *ctns^-/-^* mice compared to vehicle-treated KO animals (Figure 6K).

### Pepstatin A induces Sipa1 expression and regulates Fibronectin/STAT3/JunB pathway in *ctns^-/-^* osteoblasts

**Figure 7:**
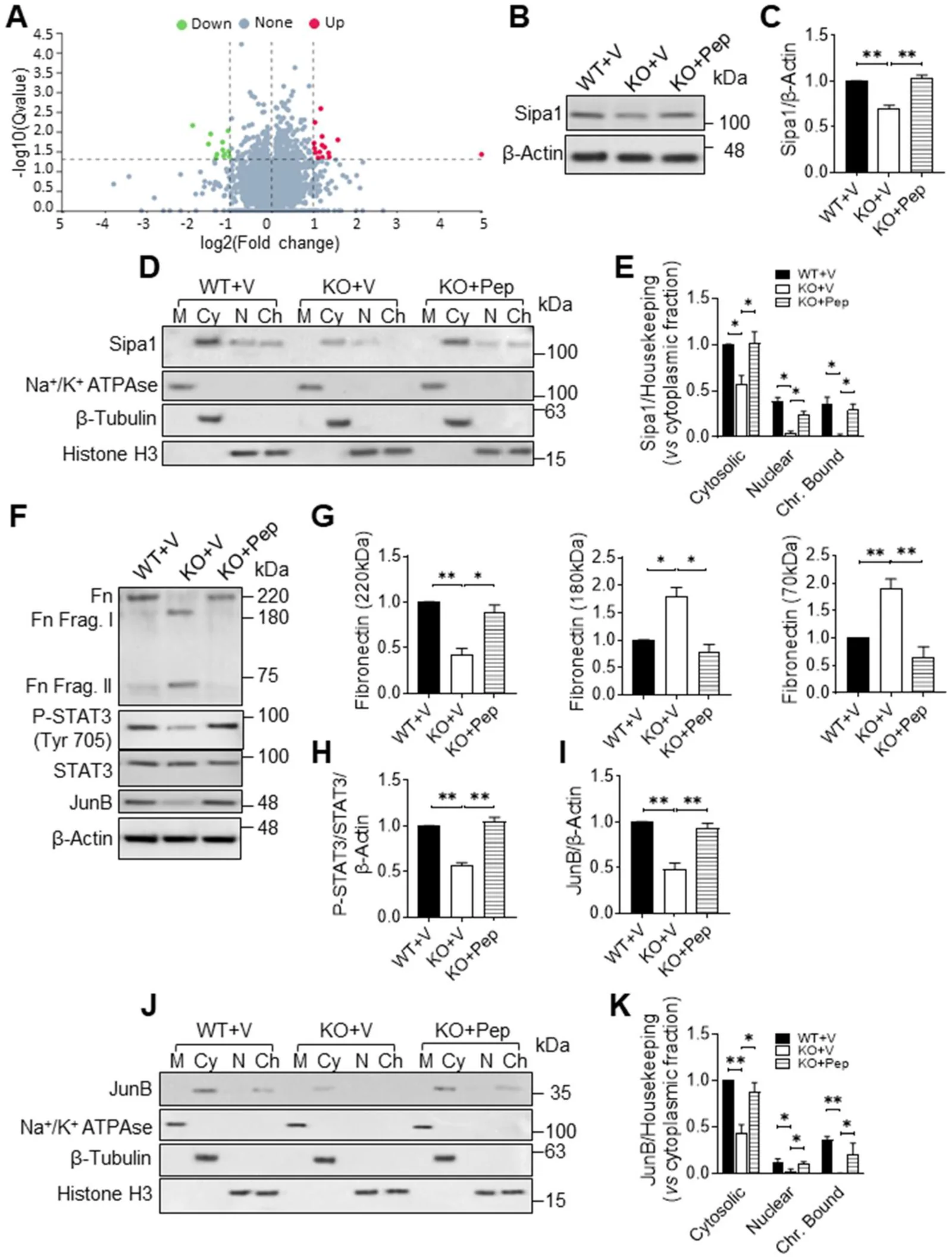
** Molecular pathway regulated by Pepstatin A in *ctns^-/-^* osteoblasts. (A)** Graphical representation of the differentially expressed proteins (DEPs) comparing Pepstatin A- or vehicle-treated *ctns^-/-^* osteoblasts. Dashed gray lines indicate the set limits of log_2_(fold change > |2|) and statistically significant values (-log_10_Q-value < 0.05). Red and green dots indicate the changes for significant DEPs (over-expression and under-expression in Pepstatin A-treated *ctns^-/-^* osteoblasts, respectively). **(B)** Western blot showing the protein levels of Sipa1 and** (C)** densitometric analysis of its levels from (**B**) normalized against β-Actin. **(D)** Western blot showing the sub-cellular localization of protein levels of Sipa1 (M: membrane; Cy: cytosolic; N: nuclear; Ch: chromatin-bound) and **(E)** densitometric analysis of the protein levels from (**D**) normalized against either Na^+^/K^+^ ATPase, β-tubulin and histone H3, for the membrane, cytosolic, and nuclear/chromatin-bound fractions, respectively, and compared to the cytosolic fraction of wild-type osteoblasts. **(F)** Representative blots of full-length Fibronectin and its fragments, STAT3 and its phosphorylated form on Tyr 705 and JunB. **(G-I)** Densitometric analysis of the protein levels from (**F**) normalized against β-Actin. **J)** Western blot showing the sub-cellular localization of protein levels of JunB and **(K)** densitometric analysis of the protein levels from (**J**) normalized against either Na+/K+ ATPase, β-tubulin and histone H3, and compared to the cytosolic fraction of wild-type osteoblasts. Values are reported as mean±SEM of at least 3 independent cell cultures. *p<0.05, **p<0.01.

To deeply investigate the molecular mechanisms regulated by Pepstatin A treatment in *ctns^-/-^* osteoblasts, we performed a second untargeted mass spectometry-based proteomic analysis, revealing 27 differentially expressed proteins in Pepstatin A-treated *ctns^-/-^* osteoblasts (Figure 7A). More specifically, 12 proteins were downregulated and 15 were upregulated. The most upregulated protein was Signal-induced proliferation-associated 1 (Sipa1), a RAP1 GTPase-activating protein. As shown in Figure 7B-C, Sipa1 was downregulated in *ctns^-/-^* osteoblasts compared to the wild-type cells. Since Sipa1 translocates in response to environmental stimuli from the cytoplasm to the nucleus 33, we evaluated its subcellular distribution by Western blot analysis. Sipa1 levels were reduced in cytoplasmic, nuclear and chromatin-bound fractions in vehicle-treated *ctns^-/-^* cells (Figure 7D-E). Pepstatin A was able to rescue Sipa1 total levels and its subcellular distribution reaching values similar to those observed in wild-type cells (Figure 7B-E). As Sipa1 has been reported to be regulated by Fibronectin 34, a known substrate of Cathepsin D 35, we next evaluated the expression of full-length Fibronectin and its proteolytic fragments in wild-type and *ctns^-/-^* osteoblasts treated with Pepstatin A or vehicle. Consistent with increased Cathepsin D activity, *ctns^-/-^* cells exhibited enhanced Fibronectin proteolysis, resulting in the generation of fragments of approximately 180 kDa and 70 kDa (Figure 7F-G). Fibronectin regulates Sipa1 trafficking between the cytoplasm and the nucleus 34, promoting STAT3 phosphorylation 36 and thereby positively regulating osteoblast differentiation and activity 37. No differences in total STAT3 expression were observed between groups (Figure 7F). However, *ctns^-/-^* osteoblasts displayed a 43.5% reduction in STAT3 phosphorylation on Tyr 705 (Figure 7F, H). STAT3 transcriptional activity is also known to regulate JunB, that can be shuttled between the cytoplasm and the nucleus acting as a transcription factor and regulating osteoblast physiology 38. JunB expression was reduced by 53% in KO cells treated with vehicle (Figure 7F, I). Pepstatin A restored Fibronectin proteolysis and STAT3 phosphorylation to levels comparable to those of wild-type osteoblasts (Figure 7F-H). Consistently, Pepstatin A-treated *ctns^-/-^* osteoblasts exhibited the normalization of JunB levels and localization, as demonstrated by MS analysis (Supplementary Table S3) and Western blot analysis (Figure 7F, I-K), thus showing upregulation of its expression compared to vehicle-treated KO cells.

## Discussion

Bone complications are well known to physicians who manage patients with nephropathic cystinosis and can significantly impact linear growth and quality of life. In our previous study, we demonstrated that the reduced bone mass in cystinosis results not only secondary from renal dysfunction but also from a primary defect in bone cells caused by cystinosin deficiency 15. Specifically, we observed that osteoblasts isolated from *ctns^-/-^* mice exhibited reduced alkaline phosphatase and mineralization activities. Concurrently, bone marrow-derived macrophages from *ctns^-/-^* mice were characterized by reduced osteoclastogenesis and bone resorption activity. Collectively, these results revealed low bone remodeling activity in *ctns^-/-^* mice 15.

Cysteamine is currently the only available cystine-depleting therapy for cystinosis. By reducing intra-lysosomal cystine accumulation, it prevents crystal formation and delays tissue damage as well as kidney dysfunction when initiated early in life 39. However, cysteamine cannot cure renal Fanconi syndrome and signs of toxicity have also been reported within the therapeutic dose range 18.

Notably, several patients with nephropathic cystinosis develop rickets secondary to the proximal renal tubulopathy. While rickets progressively resolves after treating patients with phosphate supplements and active vitamin D analogues, some patients can develop skin abnormalities and clinically relevant bone deformities, despite having normal calcium and phosphate metabolism 18. In the present study, we demonstrated that cysteamine treatment at the minimum concentration required to halve intralysosomal cystine content in *ctns^-/-^* osteoblasts significantly impaired their differentiation and mineralization capacity *in vitro*. In particular, cysteamine further reduced alkaline phosphatase enzymatic activity in mature *ctns^-/-^* osteoblasts, despite a modest increase in cell viability after 24 hours of treatment. Moreover, cysteamine exhibited a detrimental effect on bone marrow stromal cells, as evidenced by a marked reduction in colony-forming unit capacity and a consequent decrease in the number of ALP-positive colonies. These findings are consistent with the previous observations by Claramunt-Taberner *et al.* who reported a trend toward reduced expression of osteogenic markers in wild-type mice-derived MSC (Mesenchymal Stromal Cells) differentiated into osteoblasts in the presence of 200 µM cysteamine 19. Importantly, the authors showed that the pro-osteogenic effects of low dose cysteamine (10-50 µM) were lost at higher doses 19.

Our data further support the hypothesis that cysteamine interferes with bone matrix deposition, and are in agreement with the findings by Chen *et al*., who demonstrated mineralization defects in zebrafish treated with cysteamine 40. This impairment may be related, at least in part, to cysteamine-induced alterations in collagen cross-linking 18, 41. In addition, we have shown in the present study that stromal cell-supported osteoclastogenesis was completely abolished following cysteamine treatment in total bone marrow *ctns^-/-^* cell cultures. Consistently, *in vivo* intraperitoneal administration of cysteamine failed to improve somatic growth and bone mass of *ctns^-/-^* animals*.*

To identify a therapeutic approach to induce anabolic stimuli in cystinosis-related bone disease, we investigated proteomic changes in *ctns^-/-^* osteoblasts. Our analysis revealed a differential expression of 38 proteins, among which Cathepsin D was markedly upregulated. Cathepsin D is one of the most abundant lysosomal aspartyl proteases involved in the degradation of mis-folded and denatured proteins as well as the regulation of proteins and enzyme activity. Dysregulation of Cathepsin D has been implicated in several pathological conditions, including Parkinson's disease, Alzheimer's disease and Huntington's disease, as well as acute kidney injury and renal fibrosis 42. Altered Cathepsin D/pro-Cathepsin D processing has also been reported in *ctns^-/-^* proximal tubular cells 43.

Importantly, abnormal expression of Cathepsin D expression has previously been associated with bone disease. Specifically, high levels of Cathepsin D were described in hypophosphatemic *hyp* mice, and *in vivo* administration of cathepsin inhibitors improved bone mineralization in this model 32, 44.

In line with these observations, our *in vitro* and *in vivo* results demonstrated that inhibition of Cathepsin D by Pepstatin A rescues the physiological bone remodeling activity and bone phenotype in cystinosis; moreover, the recovery of body weight observed in Pepstatin A-treated KO mice raises the possibility that beyond the beneficial effects on the skeletal phenotype the treatment could involve systemic mechanisms and has effects also in others systems first of all chondrocytes. Our *in vitro* experiments demonstrated that Pepstatin A stimulated osteoblasts, through a mechanism involving the Fibronectin/Sipa1/STAT3/JunB signaling pathway. Proteomic analysis showed that Pepstatin A stimulated the expression of Sipa1 in *ctns^-/-^* osteoblasts. Sipa1 is a RAP1 GTPase-activating protein that regulates the signaling of integrins, growth factors, and cytokines. Moreover, as reported by Xiao *et al.*, *sipa1^-/-^* mesenchymal stem cells are characterized by the reduced ability to differentiate into osteoblasts, and Sipa1 KO mice showed reduced femur bone volume 45. The effects of Sipa1 modulation could be mediated by STAT3 phosphorylation 36. Notably, we reported reduced levels of STAT3 phosphorylation in *ctns^-/-^* osteoblasts that was rescued following Pepstatin A treatment. STAT3 is required for osteoblast differentiation and bone formation 46 and regulates the expression of JunB that we found to be downregulated in *ctns^-/-^* cells. The expression of JunB was restored to control levels following Pepstatin A treatment. JunB plays a significant role in the osteogenic differentiation of mesenchymal cells. Conditional KO of JunB in mice causes osteopenia secondary to combined osteoblast and osteoclast defects 38.

In conclusion, our findings identified Cathepsin D inhibition as a potential strategy to counteract the growth and skeletal defects associated with cystinosis by targeting the Fibronectin/Sipa1/STAT3/JunB pathway. However, since Cathepsin D is a key lysosomal protease involved in protein turnover and autophagic flux, we cannot exclude the possibility that some of the observed effects may be mediated, at least in part, by modulation of these pathways. Notably, to the best of our knowledge, this approach is the first therapeutic strategy shown to directly and positively impact the bone phenotype in cystinosis. However, Pepstatin A was not able to reduce intracellular cysteine content, suggesting that combined or sequential Pepstatin A/Cysteamine treatment should be investigated in future studies. In addition, our *in vivo* experiments were performed in pre-pubertal mice treated from postnatal day 4 to day 30, primarily focusing on the effects of treatment on bone growth and modeling. Future experiments will be required to evaluate the efficacy of this approach in adult mice, characterizing its impact on the bone remodeling activity.

Overall, these findings provide a proof of concept for the development of novel therapeutic strategies specifically targeting the skeletal complications of cystinosis.

## Supplementary Material

Supplementary figures and tables.

## Figures and Tables

**Table 1:** List and Sequence of Primers

Gene	Forward	Reverse
*runx2*	5′-CCCAGCCACCTTTACCTACA-3′	5′-TATGGAGTGCTGCTGGTCTG-3′
*sp7*	5′-ACCTTTCGTCTTCCTGAGCTT-3′	5′-CTGGCGCATAGGGGTTAAGT-3′
*alp*	5′-GGACAGGACACACACACACA-3′	5′-CAAACAGGAGAGCCACTTCA-3′
*col1a2*	5′-CCGTGCTTCTCAGAACATCA-3′	5′-GAGCAGCCATCGACTAGGAC-3′
*gapdh*	5′-GTTCCTACCCCCAATGTGT-3′	5′-GTGAGGGAGATCCTCAGTG-3′
*tracp*	5′-GAGAACGGTGTGGGCTATGT-3′	5′-CTGTGGGATCAGTTGGTGTG-3′
*mmp9*	5′-TGAATCAGCTGGCTTTTGTG-3′	5′-GTGGATAGCTCGGTGGTGTT-3′
*ctsk*	5′-GTAGCCACGCTTCCTATCCG-3′	5′-CCTCCAGGTTATGGGCAGAG-3′

**Table 2:** Microarchitectural parameters of distal femurs and femoral mid-diaphysis from WT and KO mice treated with Pepstatin A (Pep)/Vehicle (V).

	WT+V	KO+V	KO+Pep
**Trabecular Bone**			
**Conn. D (mm^-3^)**	244±14.4	192.9±9.8^*^	232.3±13.9^#^
**SMI**	2.08±0.05	2.23±0.03^*^	1.89±0.07^###^
**Cortical Bone**			
**Ma.Ar. (mm^2^)**	1.263±0.026	1.054±0.023^****^	1.219±0.033^##^
**Tt.Ar. (mm^2^)**	1.698±0.031	1.374±0.027^****^	1.647±0.046^###^
**Cort. TMD (mgHA/cm^3^)**	1002±4.59	990±9.38	1013±7.49
**pMOI (mm^4^)**	0.210±0.007	0.126±0.005^****^	0.200±0.011^####^
**I max (mm^4^)**	0.131±0.004	0.077±0.009^****^	0.125±0.006^####^
**I min (mm^4^)**	0.079±0.003	0.050±0.002^****^	0.076±0.004^###^

Values are reported as means±SEM. *p < 0.05, ****p < 0.0001 *vs* wild-type mice treated with vehicle; #p < 0.05, ##p < 0.01, ###p < 0.001, ####p < 0.0001*vs ctns^-/-^* mice treated with vehicle. Conn.D, connectivity density; SMI, structural model index; Ma.Ar, medullary area; Tt.Ar, total area; Cort.TMD, cortical tissue mineral density; pMOI, polar moment of inertia; I max, maximum moment of inertia; I min, minimum moment of inertia.
