# Supplementary material for: From Cysteamine to Pepstatin A: Targeting Cathepsin D To Improve Bone Health in Cystinosis
Source: Int J Biol Sci. 2026 Aug 11;22(13):7209–24. doi: 10.7150/ijbs.135791 (PMC13540694; doi:10.7150/ijbs.135791)
Supplement: Supplementary file 1 [file ijbsv22p7209s1.pdf]

Supplementary files

Table S1. Differentially expressed proteins (DEPs) between KO and WT osteoblasts

Majority protein IDs	Protein name	Gene name	Log_{10} (Ratio KO/WT)	$-Log_{10}$ Student's T-test *p*-value	# Peptides	Sequence coverage [%]	Mol. weight [kDa]	Score
**Q07113**	Cation-independent mannose-6-phosphate receptor	Igf2r	2.376	1.83441	8	4	273.81	24.45
**P29268**	Connective tissue growth factor	Ctgf	2.06693	1.55464	11	38.8	37.824	13.588
**Q99J56**	Derlin-1	Derl1	1.42834	1.30125	2	7.6	28.834	3.4049
**P49945; P29391**	Ferritin light chain 2; Ferritin light chain 1	Ftl2; Ftl1	1.41749	1.88586	1	8.7	20.842	3.3979
**O88587**	Catechol O-methyltransferase	Comt	0.691961	1.42105	3	18.1	29.486	8.7854
**Q9CQV6**	Microtubule-associated proteins 1A/1B light chain 3B	Map1lc3b	0.640702	1.43313	1	11.2	14.617	2.7413
**P57716**	Nicastrin	Ncstn	0.586488	1.55375	8	14.8	78.491	7.9425
**P18242**	Cathepsin D	Ctsd	0.473705	1.40413	11	33.4	44.953	235.69
**O35405**	Phospholipase D3	Pld3	0.434557	1.43242	3	6.4	54.388	4.9221
**O88653**	Ragulator complex protein LAMTOR3	Lamtor3	0.430358	1.57383	1	8.9	13.553	2.0685
**P03958**	Adenosine deaminase	Ada	0.409483	1.89906	2	8	39.991	3.306
**Q9WV54**	Acid ceramidase;Acid ceramidase subunit alpha;Acid ceramidase subunit beta	Asah1	0.36557	1.46108	11	33.5	44.669	27.28
**Q9D281**	Protein Noxp20	Fam114a1	0.292821	1.36667	7	16	61.012	19.19
**Q8K4Z3**	NAD(P)H-hydrate epimerase	Apoa1bp	0.234261	1.6817	4	24.5	30.972	28.783
**Q9R0N0**	Galactokinase	Galk1	0.146313	1.31582	8	23.5	42.295	23.569
**P20108**	Thioredoxin-dependent peroxide reductase, mitochondrial	Prdx3	0.132413	1.57848	6	33.5	28.127	36.369
**P68254**	14-3-3 protein theta	Ywhaq	0.116844	1.44759	12	50.6	27.778	267.1
**Q60710**	Deoxynucleoside triphosphate triphosphohydrolase SAMHD1	Samhd1	0.0739272	1.66242	6	13.6	72.65	12.747
**Q61316**	Heat shock 70 kDa protein 4	Hspa4	-0.0681311	1.3084	21	34.4	94.132	163.02
**P61750**	ADP-ribosylation factor 4	Arf4	-0.116745	1.49901	8	54.4	20.396	63.031

**P26041**	Moesin	Msn	-0.119288	1.51716	26	43.7	67.766	280.42
**Q9CZU6**	Citrate synthase, mitochondrial	Cs	-0.138243	1.67977	11	27.2	51.736	58.705
**O55143**	Sarcoplasmic/endoplasmic reticulum calcium ATPase 2	Atp2a2	-0.154687	2.32774	18	22	114.86	58.123
**P17918**	Proliferating cell nuclear antigen	Pcna	-0.156385	1.36056	6	33	28.785	18.645
**P00493**	Hypoxanthine-guanine phosphoribosyltransferase	Hprt1	-0.171571	2.11466	7	37.6	24.57	26.975
**Q9Z2Q6**	Septin-5	Sept5	-0.181515	1.67854	4	16.5	42.747	7.3309
**Q99MN1**	Lysine--tRNA ligase	Kars	-0.188368	1.64474	9	17	67.839	12.438
**P62717**	60S ribosomal protein L18a	Rpl18a	-0.193097	1.39496	6	34.7	20.732	20.548
**Q01768**	Nucleoside diphosphate kinase B	Nme2	-0.212598	1.58199	10	78.9	17.363	180.07
**Q9CR16**	Peptidyl-prolyl cis-trans isomerase D	Ppid	-0.255185	1.69854	3	15.1	40.742	3.0592
**Q9WTI7**	Unconventional myosin-Ic	Myo1c	-0.260151	1.83578	25	30.3	121.94	70.687
**P40142**	Transketolase	Tkt	-0.276361	2.5033	19	40.9	67.63	238.94
**P14069**	Protein S100-A6	S100a6	-0.289586	1.55266	2	16.9	10.051	38.083
**Q8R422**	CD109 antigen	Cd109	-0.361866	1.5944	8	7.7	161.66	15.029
**Q80UM3**	N-alpha-acetyltransferase 15, NatA auxiliary subunit	Naa15	-0.36825	1.35176	4	5.5	100.96	4.3588
**P35293**	Ras-related protein Rab-18	Rab18	-0.708262	1.45724	7	41.7	23.035	11.649
**P62878**	E3 ubiquitin-protein ligase RBX1; E3 ubiquitin-protein ligase RBX1, N-terminally processed	Rbx1	-1.56738	1.74006	2	24.1	12.274	13.829
**Q9CQN3**	Mitochondrial import receptor subunit TOM6 homolog	Tomm6	-1.94854	2.94063	1	18.9	7.8648	2.3484

In the table are listed the following DEPs' parameters: the unique protein sequence identifiers (IDs) of those DEPs that have at least half of the peptides that the leading protein has (Majority protein IDs), the Protein name and the Gene Name the protein is associated with, provided by UniProtKB/Swiss-Prot Knowledgebase; the ratio of expression (Log_{10} (Ratio KO/WT)); the negative logarithm of the *p*-value from the Student's T-test comparing the two conditions (KO *vs* WT) (-Log_{10} Student's T-test *p*-value); the total number of identified peptide sequences matching to the protein (# Peptides); the percentage of the DEP sequence that is covered by the identified peptides (Sequence coverage [%]); the molecular weight mass of the identified protein (Mol. weight [kDa]); MaxQuant's search engine Andromeda Score for the best associated MS/MS spectrum.

Table S2. Gene Ontology (GO) terms and pathways associated with the differentially expressed proteins (DEPs) between KO and WT mice osteoblasts, as obtained by the PANTHER Over-representation Test

	**PANTHER Category**	***Mus musculus* reference list (total # 22296)**	**# Protein input (38 DEPs)**	**# Protein input (expected)**	**Over/under expression**	**Fold enrichment**	***p*-value**	**FDR (< 0.05)**
**Molecular function**	protein C-terminus binding (GO:0008022)	232	5	0.4	+	12.65	4.87E-05	3.18E-02
	purine nucleoside binding (GO:0001883)	356	6	0.61	+	9.89	3.10E-05	4.72E-02
	nucleoside binding (GO:0001882)	365	6	0.62	+	9.64	3.55E-05	4.06E-02
	identical protein binding (GO:0042802)	1861	12	3.17	+	3.78	4.01E-05	3.66E-02
	small molecule binding (GO:0036094)	2387	15	4.07	+	3.69	3.95E-06	1.80E-02
	nucleotide binding (GO:0000166)	2006	12	3.42	+	3.51	8.32E-05	4.75E-02
	nucleoside phosphate binding (GO:1901265)	2006	12	3.42	+	3.51	8.32E-05	4.23E-02
	catalytic activity (GO:0003824)	5712	23	9.74	+	2.36	5.89E-06	1.35E-02
	protein binding (GO:0005515)	8991	28	15.32	+	1.83	4.06E-05	3.09E-02
**Slim Biological Process**	organonitrogen compound catabolic process (GO:1901565)	105	4	0.18	+	22.35	3.49E-05	6.22E-02
	organonitrogen compound metabolic process (GO:1901564)	237	5	0.4	+	12.38	5.37E-05	4.79E-02
**Cellular component**	autolysosome (GO:0044754)	12	2	0.02	+	97.79	2.53E-04	2.54E-02
	secondary lysosome (GO:0005767)	15	2	0.03	+	78.23	3.77E-04	3.61E-02
	ruffle (GO:0001726)	144	4	0.25	+	16.3	1.15E-04	1.44E-02
	myelin sheath (GO:0043209)	214	4	0.36	+	10.97	5.02E-04	4.39E-02
	lysosome (GO:0005764)	419	7	0.71	+	9.8	6.62E-06	1.21E-03
	lytic vacuole (GO:0000323)	420	7	0.72	+	9.78	6.72E-06	1.13E-03
	vacuole (GO:0005773)	511	7	0.87	+	8.04	2.35E-05	3.62E-03
	vesicle (GO:0031982)	1925	12	3.28	+	3.66	5.58E-05	7.47E-03

	mitochondrion (GO:0005739)	1778	11	3.03	+	3.63	1.34E-04	1.58E-02
	cytosol (GO:0005829)	3452	21	5.88	+	3.57	1.94E-08	1.95E-05
	endomembrane system (GO:0012505)	3756	16	6.4	+	2.5	2.20E-04	2.33E-02
	cytoplasmic part (GO:0044444)	8362	35	14.25	+	2.46	2.80E-12	5.62E-09
	intracellular organelle part (GO:0044446)	7217	24	12.3	+	1.95	1.58E-04	1.77E-02
	cytoplasm (GO:0005737)	10822	35	18.44	+	1.9	1.98E-08	1.33E-05
	organelle part (GO:0044422)	7570	24	12.9	+	1.86	4.11E-04	3.76E-02
	membrane-bounded organelle (GO:0043227)	10867	33	18.52	+	1.78	1.65E-06	5.54E-04
	intracellular membrane-bounded organelle (GO:0043231)	10169	30	17.33	+	1.73	4.98E-05	7.14E-03
	intracellular organelle (GO:0043229)	11823	34	20.15	+	1.69	2.13E-06	6.12E-04
	organelle (GO:0043226)	12119	34	20.65	+	1.65	4.73E-06	1.19E-03
	intracellular (GO:0005622)	13544	37	23.08	+	1.6	2.62E-07	1.32E-04
	intracellular part (GO:0044424)	13544	37	23.08	+	1.6	2.62E-07	1.05E-04
	cell part (GO:0044464)	15974	38	27.23	+	1.4	4.90E-06	1.09E-03
	cell (GO:0005623)	15976	38	27.23	+	1.4	4.91E-06	9.86E-04
**Reactome pathways**	Metabolism of nucleotides (R-MMU-15869)	97	4	0.17	+	24.2	2.58E-05	2.09E-02
	Neutrophil degranulation (R-MMU-6798695)	553	7	0.94	+	7.43	3.86E-05	2.08E-02
	Innate Immune System (R-MMU-168249)	1039	9	1.77	+	5.08	5.07E-05	2.05E-02
	Unclassified (UNCLASSIFIED)	12915	8	22.01	-	0.36	7.43E-06	1.20E-02

The input expected value is the number of proteins it would be expected in the test list for a particular PANTHER category, based on the reference list. Over-representation (+) indicates the presence of more observed proteins in the input list than expected. The fold enrichment is a measure of the frequency of a feature in the input list compared to what would be expected by random chance in a larger background. It is calculated as the ratio of the observed frequency to the expected frequency.

Table S3. Differentially expressed proteins (DEPs) between Pepstatin A (Pep) and vehicle (V) treated- KO osteoblasts.

Protein ID	Gene name	Protein name	Gene ID	Log_2 (FC Pep/V)	q-value
**P46062**	Sipa1	Signal-induced proliferation-associated protein 1	20469	5.038635	0.037601785
**P09450**	Junb	Transcription factor JunB	16477	1.590511	0.016116659
**Q5DTK1**	Chss3	Chondroitin sulfate synthase 3	78923	1.384066	0.043477807
**Q6ZQ88**	Kdm1a	Lysine-specific histone demethylase 1A	99982	1.368524	0.034605251
**P60521**	Gbrl2	Gamma-aminobutyric acid receptor-associated protein-like 2	93739	1.274071	0.024688524
**Q8VEE1**	Lmcd1	LIM and cysteine-rich domains protein 1	30937	1.219759	0.044616176
**Q8VC65**	Nrm	Nurim	106582	1.213827	0.022053735
**Q8VD76**	Tf2h3	General transcription factor IIH subunit 3	209357	1.213007	0.013221426
**Q3U2C5**	Rn149	E3 ubiquitin-protein ligase RNF149	67702	1.180321	0.002609583
**E9Q5G3**	Kif23	Kinesin-like protein KIF23	71819	1.158557	0.032635521
**Q91XC9**	Pex16	Peroxisomal membrane protein PEX16	18633	1.116526	0.04580868
**O08609**	Mlx	Max-like protein X	21428	1.057887	0.033884702
**Q91W52**	Tmm19	Transmembrane protein 19	67226	1.041509	0.005820667
**Q9QWH1**	Phc2	Polyhomeotic-like protein 2	54383	1.031098	0.026504006
**P59110**	Senp1	Sentrin-specific protease 1		1.020518	0.019472084
**Q80WV3**	Chst2	Carbohydrate sulfotransferase 2	54371	-1.013907	0.042579732
**Q3ULJ0**	Gpd1l	Glycerol-3-phosphate dehydrogenase 1-like protein	333433	-1.028411	0.031375259
**Q8R060**	Zwilc	Protein zwilch homolog	68014	-1.041875	0.009508835
**Q6P9J9**	Ano6	Anoctamin-6	105722	-1.12918	0.037178
**Q8CGC4**	Ls14b	Protein LSM14 homolog B	241846	-1.147407	0.041468128
**O70201**	Birc5	Baculoviral IAP repeat-containing protein 5	11799	-1.17025	0.028857874
**Q6PAL8**	Den5a	DENN domain-containing protein 5A	19347	-1.177381	0.018427412
**Q60969**	Styx	Serine/threonine/tyrosine-interacting protein	56291	-1.310415	0.037274055
**Q8BJL1**	Fbxo30	F-box only protein 30	71865	-1.335167	0.045912647
**Q8VCM7**	Fgg	Fibrinogen gamma chain	99571	-1.460121	0.011425654
**Q80YA3**	Ddhd1	Phospholipase DDHD1	114874	-1.513901	0.020552961

**Q8CE33**	Klhl11	Kelch-like protein 11	217194	-1.892887	0.006976772

In the table are listed the following DEPs' parameters: the unique protein sequence identifiers (Protein IDs), the Gene Name the protein is associated with, the protein name provided by UniProtKB/Swiss-Prot Knowledgebase; the unique gene identifier (Gene ID) assigned by the NCBI Entrez Gene database; the ratio of expression [Log_2(Fold Change)]; and the p-values adjusted for multiple comparisons using the Benjamini-Hochberg False Discovery Rate procedure (q-value).

Supplementary Figure 1: *In vitro* effect of cysteamine on *ctns*$^{-/-}$ osteoblasts isolated from long bones. Osteoblasts were isolated from femurs and tibias of neonatal WT and KO mice and treated with cysteamine (Cys, 100µM) or Vehicle (V). **A)** Representative pictures (Original magnification 10X) and **B)** densitometric analysis of ALP staining. **C)** Cell viability assessed by XTT assay. Data are reported as mean±SEM of at least 3 independent cell cultures. *p<0.05, **p<0.01, ***p<0.001.

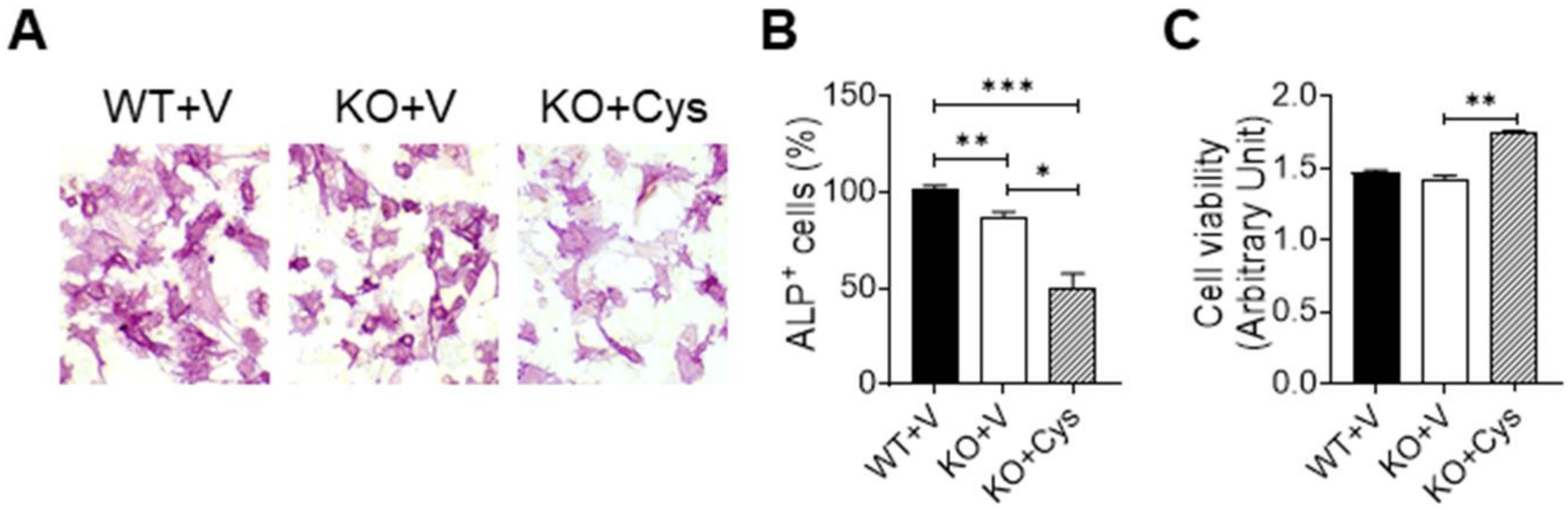

Supplementary Figure 1

Supplementary Figure 2: Effect of cysteamine treatment *in vivo*. WT and KO mice treated with vehicle (V) or cysteamine (Cys, 120 µg/g/day). **A-C)** Weight of **A)** spleen, **B)** liver and **C)** kidney of wild-type and *ctns*$^{-/-}$ mice treated with vehicle or with cysteamine. Data are reported as mean±SEM. *$p<0.05$. **D)** Representative pictures of Hematoxylin/eosin staining of spleen, liver and kidney. Original Magnification 10X. Scale bar 500 µm.

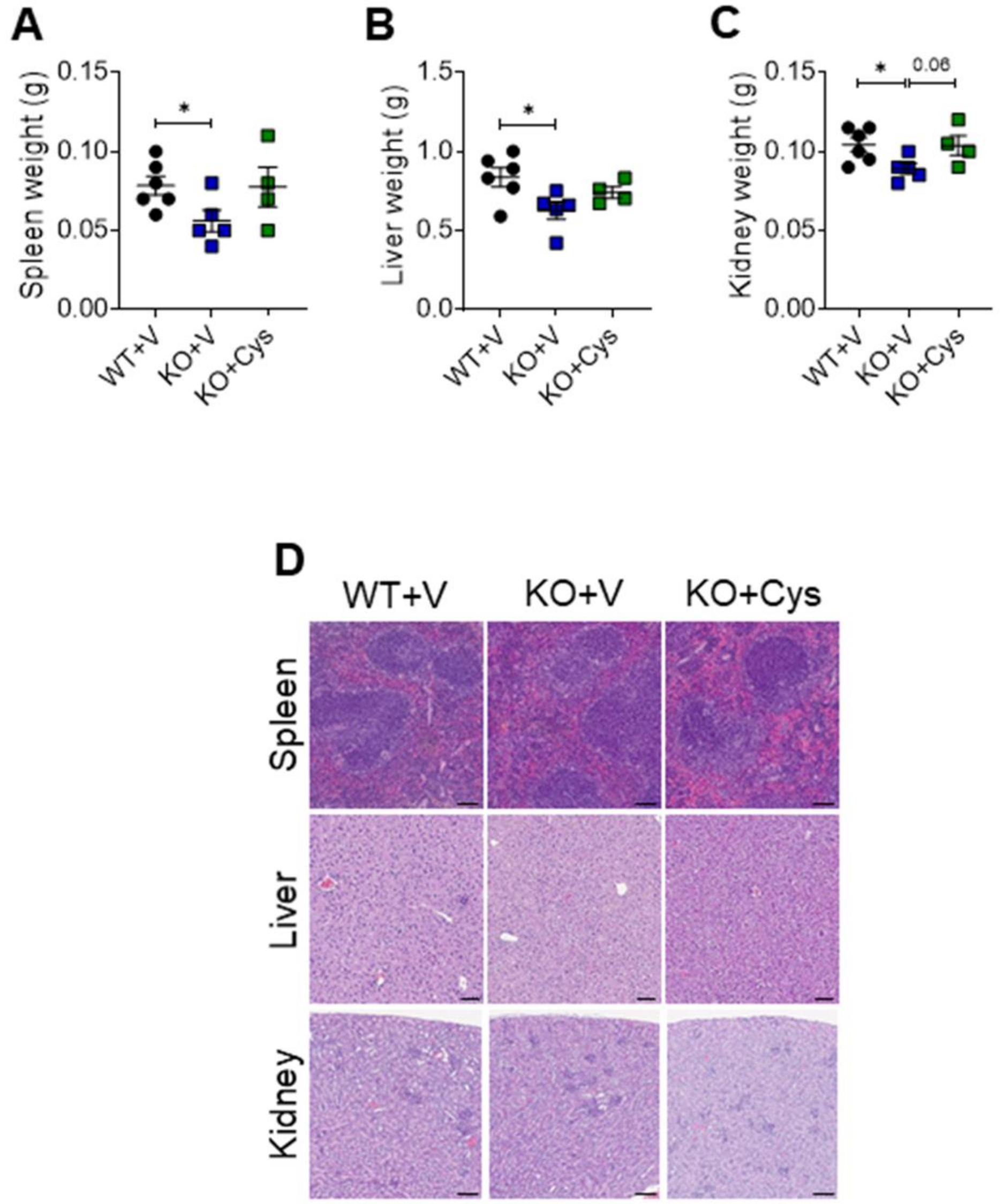

Supplementary Figure 2
